# A CsPb_0.95_Ni_0.05_Br_3_ NCs-based fluorescence sensor for rapidly and accurately evaluating trace water in edible oils along with the structure destruction and dissolution

**DOI:** 10.1016/j.fochx.2025.102196

**Published:** 2025-01-16

**Authors:** Qin Ye, Penghao Zhu, Xianghe Meng, Jie Li, Yuanchao Lu

**Affiliations:** a Institute of Food Sciences, Zhejiang, Academy of Agricultural Sciences, Hangzhou 310014, China; b College of Food Science and Technology, Zhejiang University of Technology, Hangzhou 310014, China; c College of Chemistry and Materials Engineering, Zhejiang A&F University, Hangzhou 311300, China

**Keywords:** CsPb_0,95_Ni_0,05_Br_3_ NCs, Water content, Edible oils, Fluorescence sensors, Structure destruction and dissolution

## Abstract

Metal ions with smaller radii than Pb^2+^ can stabilize CsPbBr_3_ NCs' cubic structure by lattice shrinkage, but lacking sensing research. Herein, Ni-substituting CsPbBr_3_ NCs were prepared to rapidly and accurately detect water content (WC) in edible oils. CsPb_0.95_Ni_0.05_Br_3_ NCs had the highest fluorescence intensity, approximately 125 % of CsPbBr_3_ NCs. The results displayed that CsPb_0.95_Ni_0.05_Br_3_ NCs were uniformly quadrilateral crystalline packing (8.78 ± 0.28 nm particle size) with inter-planar distances of 0.41, 0.33, and 0.29 nm. Given the fluorescence quenching behavior, a superior linear curve between fluorescence-decreased ratio and WC was established within 0–3 ‰ (*v*/v) and a detection limitation of 0.042 ‰. Furthermore, excellent precision and accuracy were verified in various oils with a relative error of 2.06 %. It was suggested that water destroyed and dissolved CsPb_0.95_Ni_0.05_Br_3_ NCs' crystal structure to induce fluorescence quenching. Thus, Pb-site substitutions of CsPbBr_3_ NCs enhanced the sensing performance, enlightening other elements-substituted CsPbBr_3_ NCs for sensing.

## Highlights

• CsPb_0.95_Ni_0.05_Br_3_ NCs possessed the highest PL intensity (125 % of CsPbBr_3_ NCs).
• CsPb_0.95_Ni_0.05_Br_3_ NCs were 8.78 ± 0.28 nm and quadrilateral crystalline packing.
• A CsPb_0.95_Ni_0.05_Br_3_ NCs-based sensor was to access WC within 0–3 ‰ and a 0.042 ‰ LOD.
• Trace water destroyed and dissolved the crystal structure to decrease FL intensity.
• As-proposed method can access WC in various edible oils with an RSD of 2.06 %.

## 1 Introduction

Generally, edible oils deteriorate severely during processing and storage, leading to the decrease of quality, nutrition and safety (da Silva et al., 2022). It is widely regarded that water content (WC, or water activity) in edible oils, as one of the crucial factors in the deterioration rate, is suggested to be controlled within a reasonable range (0.05–0.2 %, *v*/v, GB/T 1535–2017) (Jedrkiewicz et al., 2016; Majchrzak et al., 2018; Meng et al., 2012; Valantina, 2021; Xie et al., 2018). It is believed that high WC in edible oils would likely increase the dissolved oxygen content and then accelerate oil oxidation rancidity, resulting in quality degradation. Meanwhile, high WC even likely promotes the growth of microorganisms (especially *Aspergillus flavus*) in edible oils to influence food safety (Verardo, Ferioli et al., 2009). Regarding in-depth investigations and knowledges, trace water in edible oils accelerates the quality deterioration, and sensing scenes are primarily located in the bulk storage. Thus, it is of great urgency for rapidly and sensitively evaluating WC in edible oils to ensure the quality and food safety, and even improve economic value.

To date, several strategies have been proposed to evaluate WC in edible oils, including oven drying method (GB 5009.236–2016), Karl Fischer titration (KFT), gas chromatography (GC), infrared spectroscopy, etc. (Xie et al., 2018) As a conventional method, the oven drying method is simple and convenient to operate while poor sensitivity, large errors, and long time-consuming restrict the practical application. With the properties of small-sample consumption, high accuracy, and fast response, KFT is a commonly used method based on the redox reaction between iodine and sulfur dioxide to react with water under organic surroundings quantitatively. However, professional operations and high preservation requirements for KFT reagents significantly limit the rapid or in-situ evaluation (Hakonen & Beves, 2018; Liu et al., 2020). GC can accurately and sensitively evaluate WC, while expensive instrument, and professional operations and labs make it difficult to respond quickly and in situ (Jung et al., 2016). Given the rapid response and non-destruction, infrared spectroscopy establishes the dose-effect relationship between WC and water's signal (hydroxyl or hydrogen bond) (Chen, Cao, et al., 2017). Our team proposed acetonitrile extraction D_2_O-assisted Fourier transform infrared (FTIR) spectroscopy to determine trace moisture in edible oils, and the results showed that the average error was less than 3 %. Compared with the near-infrared method, as-proposed method eliminated the requirements for preparation and separation of dry oil, and was easy to operate (Ye et al., 2023). However, shortcomings, such as low model robustness, high-cost instruments, and the immense influence of oil quality factors, still existed. In this context, it is concluded that above evaluation methods make it difficult to perceive WC in edible oils rapidly and sensitively.

Recently, a novel and advanced fluorescent material, halogen metal perovskite (HMPs, especially CsPbBr_3_ NCs), has been the research hot spot due to high luminescent quantum yields, wide fluorescence-emission ranges (covering the entire visible light region), and abundant surface defects (Gollino & Pauporte, 2021; Lu et al., 2020; Lu, Tang, Yu, et al., 2024; Yang et al., 2024; Zhang et al., 2021; Zhu et al., 2020). Given the excellent optical properties, HMPs show great potentials in food safety detection, especially in the quality evaluation of edible oils. Our team prepared CsPbBr_3_@ZIF-8 and established a fluorescence sensor for evaluating acid value of edible oils (limit of detection (LOD) = 0.06 mg KOH/g, recovery = 92–101 %) (Lu, Xiong, Lin, et al., 2024). In addition, a new visual method based on CsPbBr_3_ NCs was developed to determine peroxide value in edible oils via REDOX and halogen exchange, with a LOD of 0.0034 g/100 g and a 15-min detection time (Lu, Tang, Yu, et al., 2024). Given the structural instability to high-polar solvents (especially water), CsPbBr_3_ NCs could be, in turn, applied to rapidly and sensitively evaluate WC in edible oils. Zhao et al. prepared mesoporous silica-coated CsPbBr_1.5_I_1.5_ NCs and established a ratiometric fluorescence sensor for evaluating WC in edible oils with a LOD of 0.45 % (Zhao et al., 2021). Wang et al. prepared dual-emission CsPbBr_3_ NCs by modifying with dimethyl amino terephthalate, displaying the sensitive fluorescence turn-on/off and wavelength shift to trace water in edible oils with a LOD of 0.006 % (Wang et al., 2024). Indeed, the functionality partly improves the performance of CsPbBr_3_ NCs for detecting WC in edible oils. However, the “trade-off” between stability and performance of CsPbBr_3_ NCs is still a critical factor in sensing. In solar cells, Pb-site substituting CsPbBr_3_ NCs with smaller metal ions, including Ni^2+^, Mn^2+^, Zn^2+^, Cu^2+^, improve the stability and power conversion efficiency (Deng et al., 2022; Shapiro et al., 2019). Huang et al. demonstrated that ion radiuses of Mn^2+^ and Zn^2+^ were smaller than Pb^2+^, inducing the lattice constriction and then stabilizing the cubic structure (Huang et al., 2021). Shapiro et al. revealed that Ni^2+^ substitutions to Pb^2+^ of CsPbBr_3_ NCs could enhance the power conversion efficiency of solar cells (Shapiro et al., 2019). In this context, a hypothesis is proposed that Pb-site substitution of CsPbBr_3_ NCs would balance the stability and performance and further improve the sensing performance to WC.

However, no research about Pb-site substitution of CsPbBr_3_ NCs is located in the sensing investigation, and the “balance” between the stability and performance of CsPbBr_3_ NCs in sensing still lacks research. This study investigated the sensing performance of CsPb_(1-*x*)_Ni_x_Br_3_ NCs to WC in edible oils. First of all, CsPb_(1-*x*)_Ni_x_Br_3_ NCs (x = 0–0.23) were prepared by the room-temperature synthesis method to control the particle size precisely (Akkerman et al., 2022; Zirak et al., 2019). Next, the fluorescence (FL) intensity of CsPb_(1-*x*)_Ni_x_Br_3_ NCs (x = 0–0.23) was systematically investigated to select the superior substitution ratio. Then, as-prepared CsPb_(1-*x*)_Ni_x_Br_3_ NCs were characterized with high-resolution transmission electron microscope (HRTEM), X-ray Diffraction (XRD), and X-ray photoelectron spectroscopy (XPS). Afterward, the response performance of CsPb_(1-*x*)_Ni_x_Br_3_ NCs to WC in edible oils was systematically studied to establish a standard curve and evaluate the sensing performance. Furthermore, TEM and XRD to as-reacted CsPb_(1-*x*)_Ni_x_Br_3_ NCs were performed to reveal the potential response mechanism of trace water to CsPb_(1-*x*)_Ni_x_Br_3_ NCs. Finally, the practical application of CsPb_(1-*x*)_Ni_x_Br_3_ NCs in the evaluation of WC to various edible oils was investigated. We wish to provide a rapid and sensitive strategy for evaluating WC in edible oils.

## 2 Materials and methods

### 2.1 Materials

Cs_2_CO_3_ (99.999 %), PbBr_2_ (99.999 %), NiBr_2_ (99.99 %), diisooctylphosphinic acid (DOPA, 90 %), n-octane (99 %), lecithin (>97 % from soybean), and n-hexane were provided by Aladdin Reagent Co., Ltd. (Shanghai, China). Acetone and trioctylphosphine oxide (TOPO, >90 %) were obtained from Sinopharm Chemical Reagent Co. Ltd. (Shanghai, China). All chemicals were of analytical grade without descriptions and used as received without further purifications.

### 2.2 The preparation of CsPb_(1-*x*)_Ni_x_Br_3_ NCs

Several stock solutions were prepared before synthesizing CsPb_(1-*x*)_Ni_x_Br_3_ NCs. Pb-TOPO stock solution was obtained by dispersing 1 mmol PbBr_2_ and 5 mmol TOPO into 5 mL n-octane at 120 °C and then diluting with 20 mL n-hexane. Ni-TOPO stock solution was obtained by dispersing 1 mmol NiBr_2_ and 5 mmol TOPO into 5 mL n-octane at 120 °C and then diluting with 20 mL n-hexane. Cs-DOPA stock solution was prepared by mixing 100 mg Cs_2_CO_3_, 1 mL DOPA, and 2 mL n-octane at 120 °C and then diluting with 27 mL n-hexane. TOPO stock solution was obtained by dissolving 4 mmol TOPO into 20 mL n-hexane. Lecithin stock solution was prepared by dissolving 1 g lecithin into 20 mL n-hexane. All stock solutions were filtered through a 0.45 μm filter membrane before use.

In this research, CsPbBr_3_ NCs were prepared according to a previous research about the room-temperature synthesis method with minor modifications (Akkerman et al., 2022). Briefly, 80 μL Pb-TOPO and 240 μL TOPO stock solutions were mixed with 6 mL n-hexane. Next, 40 μL Cs-DOPA stock solution was injected into the above mixture under vigorous stirring (1500 rpm). After incubating for 5 min, 40 μL lecithin stock solution was added to realize the ligand exchange. After approximately 30 s reaction, acetone (about 18 mL), as the anti-solvent, was added. Finally, CsPbBr_3_ NCs were collected by centrifuging at 8000 rpm for 5 min and then re-dispersed into n-hexane (the concentration of CsPbBr_3_ NCs was controlled as 1 mg/mL). Through adjusting the addition of Pb-TOPO and Ni-TOPO stock solutions, CsPb_(1-*x*)_Ni_x_Br_3_ NCs (x = 0–0.23) were prepared.

### 2.3 Characterizations

To evaluate the fluorescence property, the FL spectra of CsPb_(1-*x*)_Ni_x_Br_3_ NCs (x = 0–0.23) were obtained by a multifunctional enzyme marker (SynergyH1, Bioteck, Vermont, USA). Herein, FL spectra were scanned from 400 to 600 nm under a 365 nm laser excitation. The micromorphology and surface-crystal structure of CsPb_0.95_Ni_0.05_Br_3_ NCs were investigated with a high-resolution transmission electron microscopy (HRTEM, Tecnai G2 F30 STWIN, Netherlands). Herein, particle sizes and inter-planar distances were measured with ImageJ (National Institutes of Health, USA). The crystal structure of CsPb_0.95_Ni_0.05_Br_3_ NCs was performed by a powder X-ray diffractometer (XRD, D/max-Ultima IV, Rigaku, Japan) with a recording range of 5–50° and a step size of 10°/min, operated at 40 kV and 40 mA in the X-scan mode using the Cu Kα radiation (λ = 1.541 Å). The data were analyzed with MDI Jade6 software (Materials Date, USA), and PDF#54–0752 card was regarded as the standard XRD profile of CsPbBr_3_ NCs. The X-ray photoelectron spectroscopy (XPS, AXIS Ultra DLD, Kratos, Japan) was used to evaluate the chemical interaction of CsPb_0.95_Ni_0.05_Br_3_ NCs at the recording range of 1000–0 eV. Also, high-resolution XPS spectra were recorded within the range of 745–715 eV, 150–130 eV, and 80–60 eV to display the interaction of Cs 3d, Pb 4f, and Br 3d, respectively.

### 2.4 The response performance of CsPb_0.95_Ni_0.05_Br_3_ NCs to WC in edible oils

Before investigating the response performance of CsPb_0.95_Ni_0.05_Br_3_ NCs to water, edible oils (refined *Soybean* oils from a local supermarket) were pretreated to remove trace water and eliminate the background. Briefly, *Soybean* oils were poured into the silica gel column filled with the silica gel (100–200 mesh). Next, *Soybean* oils were filtered through the silica gel column under the vacuum pump. After repeating above procedures at least three times, as-dried edible oils were collected and stored in a glass vacuum dryer for further use.

The response performance of CsPb_0.95_Ni_0.05_Br_3_ NCs to WC in edible oils was investigated according to our previous studies (Lu, Tang, Yu, et al., 2024; Lu, Xiong, Tang, et al., 2024). Briefly, as-dried edible oils were spiked with various WC (0–3 ‰, *v*/v). Then, 1 mL 1 mg/mL CsPb_0.95_Ni_0.05_Br_3_ NCs were mixed with 0.22 mL edible oils (WC 0–3 ‰, v/v). After incubating at room temperature for 10 min, FL spectra were obtained by a multifunctional enzyme marker from 400 to 600 nm under a 365 nm laser excitation. The highest FL intensity and peak wavelength were extracted to establish the standard curve.

### 2.5 Practical application

The practical application of the as-proposed CsPb_0.95_Ni_0.05_Br_3_ NCs-based method was evaluated. Herein, *Camellia* oil, *Olive* oil, *Flaxseed* oil, and *Soybean* oil were selected. Above edible oils were dried according to the method described in Section 2.4. Next, as-dried edible oils were spiked with different WC (0.5 ‰, 2 ‰, and 3 ‰) to verify detection feasibility in various edible oils. Then, FL spectra of the mixture (1 mL 1 mg/mL CsPb_0.95_Ni_0.05_Br_3_ NCs and 0.22 mL edible oils) were obtained. Finally, WC was calculated according to the standard curve (obtained from Section 2.4). Relative standard deviation (RSD) and relative errors were calculated to evaluate the precision and accuracy, respectively.

### 2.6 Data statistics

All experiments were carried out at least three times with the description of mean ± standard deviation. The significant difference (*P* < 0.05) was identified with one-way ANOVA and the Duncan test by SPSS software (SPSS 21, IBM, USA).

## 3 Results and discussion

### 3.1 Preparation and characterizations of CsPb_(1-*x*)_Ni_x_Br_3_ NCs (x = 0–0.23)

**Scheme 1:**
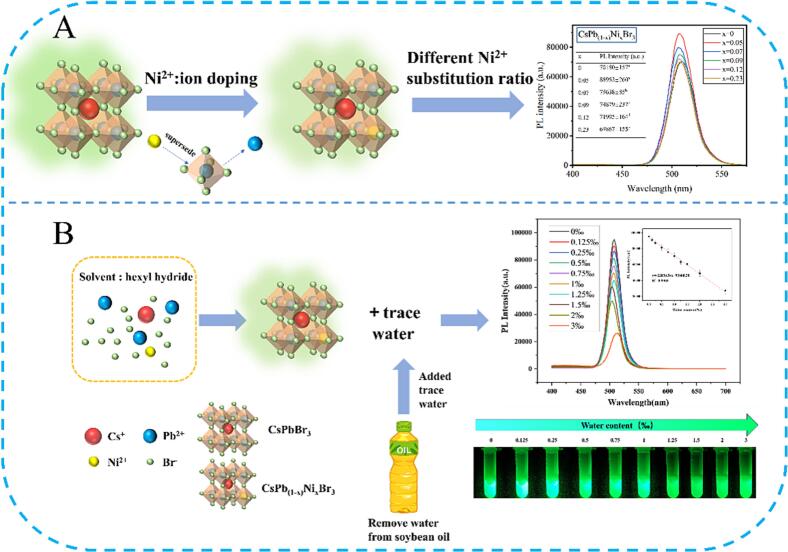
Schematic diagram of synthesis and trace water detection of CsPb_(1-*x*)_Ni_x_Br_3_ NCs (x = 0–0.23).

**Fig. 1:**
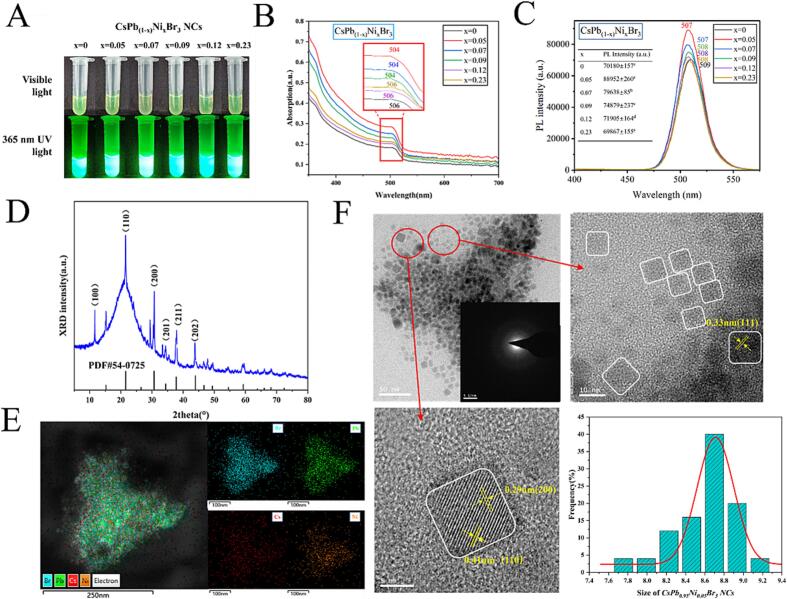
Photographs (A), UV–vis absorption (B), and FL (C) spectra of CsPbBr_3_ NCs and CsPb_(1-*x*)_Ni_x_Br_3_ NCs (x = 0.05, 0.07, 0.09, 0.12, 0.23). XRD profiles (PDF#54–0752 were regarded as the standard PDF card of CsPbBr_3_ NCs) (D), EDS elemental mapping, TEM and HRTEM images (F) of the CsPb_0.95_Ni_0.05_Br_3_ NCs (the insert figure was corresponding selected area electron diffraction (SAED) profile) and size of distribution CsPb_0.95_Ni_0.05_Br_3_ NCs.

Generally, the particle size and shape are the curtail factors to the fluorescence property of CsPbBr_3_ NCs (Akkerman et al., 2022; Kazes et al., 2021). Meanwhile, it is reported that abundant studies have demonstrated that Pb-site substitution to CsPbBr_3_ NCs with Ni^2+^ could improve solar cells' stability and power conversion efficiency (Deng et al., 2022; Shen et al., 2020). Inspired by the above perspectives, as shown in Scheme 1, CsPb_(1-*x*)_Ni_x_Br_3_ NCs (x = 0–0.23) were prepared using the room-temperature synthesis method to slow down the nucleation and growth rates and then control the particle size and shape precisely. The optical profiles of CsPb_(1-*x*)_Ni_x_Br_3_ NCs suspensions under visible light and UV irradiation (365 nm) were captured, shown in Fig. 1A. The result demonstrated that CsPb_(1-*x*)_Ni_x_Br_3_ NCs suspensions were light green under visible light and the suspension color did not significantly vary with various Ni^2+^ substitution ratios. However, CsPb_(1-*x*)_Ni_x_Br_3_ NCs suspensions presented the relatively constant green fluorescence under the 365 nm UV irradiation. Next, the UV–Vis and FL spectra of CsPb_(1-*x*)_Ni_x_Br_3_ NCs (x = 0–0.23) were obtained to select and optimize the superior Ni^2+^ substitution ratio. As shown in Fig. 1B, UV–Vis absorption peaks of CsPb_(1-*x*)_Ni_x_Br_3_ NCs were approximately 506 nm and did not differ significantly with the increase of Ni^2+^ substitution ratios. Regarding FL spectra (Fig. 1C), FL intensities of CsPb_(1-*x*)_Ni_x_Br_3_ NCs significantly increased with Ni^2+^ substitution, while the FL peak wavelength was located at 508 nm and remained unchanged. When the Ni^2+^ substitution ratio was 0.05, the FL intensity was maximum (88,952 a.u.), approximately 1.25 times of CsPbBr_3_ NCs (70,180 a.u.). Whereafter, the FL intensity decreased with the increase of Ni^2+^ substitution ratios from 0.05 to 0.23. It was believed that Ni^2+^ substitution could inhibit the non-radiative recombination and reduce the surface defects to increase the FL intensity of CsPb_(1-*x*)_Ni_x_Br_3_ NCs (Deng et al., 2022; Shen et al., 2020). In this context, x = 0.05 (CsPb_0.95_Ni_0.05_Br_3_ NCs) was regarded as the superior substitution ratio.

The crystal structure of CsPb_0.95_Ni_0.05_Br_3_ NCs was characterized by XRD, shown in Fig. 1D. The result presented that seven specific diffraction peaks at 15.34°, 21.66°, 26.90°, 30.48°, 34.34°, 37.85° and 43.89° were corresponding to the (100), (110), (111), (200), (201), (210) and (202) planes of CsPb_0.95_Ni_0.05_Br_3_ NCs, respectively, agreed with previous studies (Cheng et al., 2023; Deng et al., 2022). Micro-morphologies of CsPb_0.95_Ni_0.05_Br_3_ NCs were investigated with TEM and HRTEM. Fig. 1F showed that CsPb_0.95_Ni_0.05_Br_3_ NCs presented a uniform crystalline distribution (particle size of 8.78 ± 0.28 nm), and the crystal morphology was mainly quadrilateral. It should be noted that lattice fringes of CsPb_0.95_Ni_0.05_Br_3_ NCs were 0.41 nm, 0.33 nm, and 0.29 nm, belonging to the (110), (111), and (200) planes, respectively (Fig. 1F). The EDS mapping results (Fig. 1E) showed that Pb, Cs, and Br elements were evenly distributed, and a small amount of Ni elements appeared, close to the stoichiometric, verifying the successful preparation of CsPb_0.95_Ni_0.05_Br_3_ NCs.

**Fig. 2:**
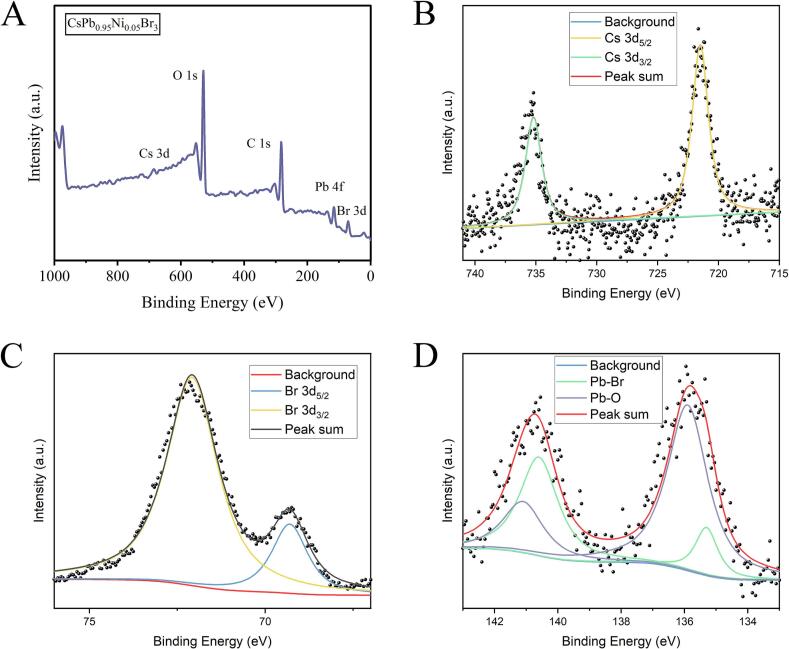
XPS patterns of CsPb0.95Ni0.05Br3 NCs. Wide XPS spectra (A), high-resolution Cs 3d (B), Br 3d (C), and Pb 4f (D) profiles.

Then, XPS profiles of CsPb_0.95_Ni_0.05_Br_3_ NCs were performed to reveal the chemical structure, shown in Fig. 2. Wide XPS spectrum (Fig. 2A) performed five prominent characteristic peaks at 685, 529, 283, 135, and 70 eV, representing Cs 3d, O 1 s, C 1 s, Pb 4f, and Br 3d, respectively (Kong et al., 2018). It should be noted that Ni element was not presented in the XPS spectrum, probably due to the trace Ni-proportion in CsPb_0.95_Ni_0.05_Br_3_ NCs (Deng et al., 2022). Regarding high-resolution Cs 3d XPS spectra (Fig. 2B), Cs 3d was divided into Cs 3d_5/2_ and Cs 3d_3/2_, and the intensity of Cs 3d_5/2_ was higher than that of Cs 3d_3/2_. Br 3d XPS spectra (Fig. 2C) were divided into Br 3d_5/2_ and Br 3d_3/2_. Meanwhile, Pb—O and Pb—Br were the divided peaks of Pb 4f spectra (Fig. 2D). In this context, CsPb_0.95_Ni_0.05_Br_3_ NCs were successfully obtained with excellent FL intensity and had great potentials to be used to sense WC in edible oils.

**Fig. 3:**
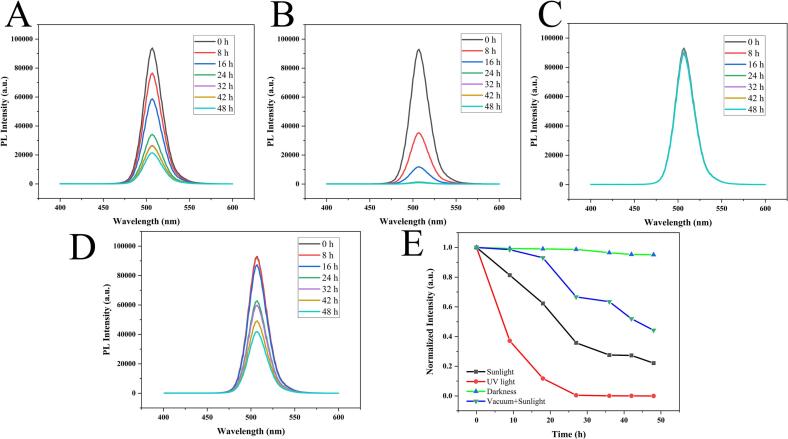
The fluorescence spectra of CsPb_0.95_Ni_0.05_Br_3_ NCs during 48 h under the four distinct environmental conditions:(A) sunlight exposure, (B) UV exposure, (C) darkness, and (D) vacuum-storage with sunlight exposure. E) the summary of FL intensities under various conditions.

The stability of CsPb_0.95_Ni_0.05_Br_3_ NCs was critical for their practical application, Therefore, the 48-h fluorescence stability of CsPb_0.95_Ni_0.05_Br_3_ NCs was investigated under four distinct environmental conditions, including sunlight exposure, UV exposure, darkness, and vacuum-storage with sunlight exposure (Fig. 3A-D). Under sunlight exposure, the FL intensity of CsPb_0.95_Ni_0.05_Br_3_ NCs was gradually decreased and maintained at approximately 21 % after 48 h (Fig. 3A). Under UV exposure, the FL intensity of CsPb_0.95_Ni_0.05_Br_3_ NCs was only maintained at approximately 5 % after 48 h (Fig. 3B). In contrast, Fig. 3C revealed that the FL peak position of CsPb_0.95_Ni_0.05_Br_3_ NCs did not change after 48 h under darkness, and the FL intensity of CsPb_0.95_Ni_0.05_Br_3_ NCs remained unchanged (Fig. 3C). While vacuum-storage with sunlight exposure maintained 43 % of the initial fluorescence intensity (Fig. 3D). The above results illustrated that the as-synthesized CsPb_0.95_Ni_0.05_Br_3_ NCs displayed outstanding stability under darkness, which can be used to guide the subsequent practical application.

### 3.2 Response performance of CsPb_0.95_Ni_0.05_Br_3_ NCs to water in edible oils

**Fig. 4:**
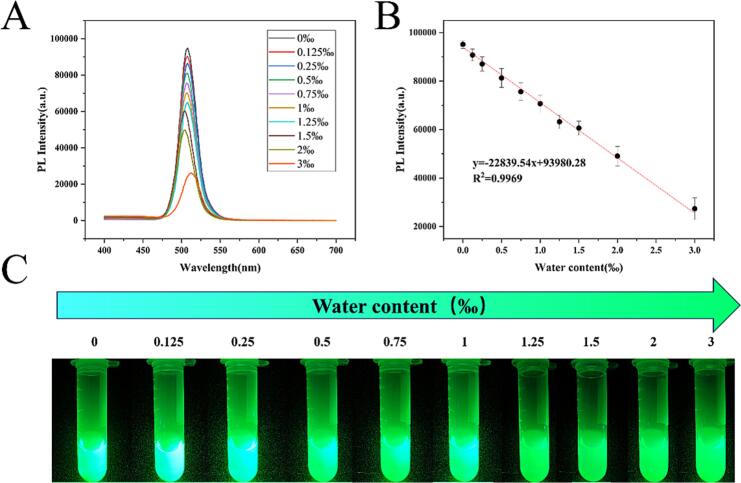
CsPb_0.95_Ni_0.05_Br_3_ NCs were exposed to edible oils with different WC (0–3‰). FL spectra (A) and fluorescence photographs (C) (at 365 nm excitation) of the mixture. Linear fitting curve (WC vs. FL intensities of CsPb_0.95_Ni_0.05_Br_3_ NCs) (B).

Generally, water could induce the crystal destruction and disintegration of CsPbBr_3_ NCs, leading to a quenching fluorescence behavior (Acharya et al., 2024). In turn, it was reasonably conjectured that WC in edible oils could be quantitatively evaluated by FL intensity of CsPb_0.95_Ni_0.05_Br_3_ NCs. Fig. 4A performed that FL intensities of CsPb_0.95_Ni_0.05_Br_3_ NCs gradually decreased with the increase of WC, indicating that high-polar water quenched the fluorescence of CsPb_0.95_Ni_0.05_Br_3_ NCs. It was believed that removing surface ligands and reducing passivated surface defects by polar water molecules led to the appearance of extensive defects and surface states, even caused the disintegration of crystal morphology, provided a practical non-radiative recombination pathway, and induced the fluorescence quenching (Acharya et al., 2024). In this context, the response performances of CsPb_0.95_Ni_0.05_Br_3_ NCs to water in edible oils were investigated with various spiked WC (0–3 ‰). Herein, 1 mL 1 mg/mL CsPb_0.95_Ni_0.05_Br_3_ NCs suspension and 0.22 mL edible oils (containing 0–3 ‰ WC) were mixed and incubated for 10 min at room temperature. Fig. 4A showed that FL intensities at 508 nm decreased with increased WC while peak position wavelength (508 nm) unchanged. When WC in edible oils was 0, the FL intensity reached the highest (94,867 a.u.). However, FL intensity significantly decreased upon exposing to trace water, and gradually decreased with the increase of WC. Under exposing to 3 ‰ WC in edible oil, the FL intensity reached the lowest (26,228 a.u.). Based on the negative correlation, an excellent linear (R^2^ = 0.9969) fitting curve was established between the FL intensity and WC (0–3 ‰) (Fig. 4B). The LOD of CsPb_0.95_Ni_0.05_Br_3_ NCs was calculated to be 0.042 ‰ based on the formula (LOD = 3*σ/k, σ and k referred to the standard deviation and slope of the linear fitting curve, respectively). Furthermore, photographs of the reaction system were captured under a 365 nm UV lamp, as shown in Fig. 4C. With the increase in WC, the fluorescence color can be recognized by the naked eye, and the fluorescence color shifted from turquoise to green. Given the consideration that LOD was much lower than the reasonable WC range (0.5–2 ‰, *v*/v) in edible oils and the working dynamic range covered the reasonable WC range (0.5–2 ‰, v/v), the as-proposed method was able to quantitatively and qualitatively access WC in edible oils. Therefore, a stable, sensitive, and reliable WC in edible oils fluorescence sensor based on CsPb_0.95_Ni_0.05_Br_3_ NCs was successfully established.

Compared to other studies (Chen, Ye, et al., 2017; Dong et al., 2016; Srivastava et al., 2011; Wang et al., 2022; Xiang et al., 2021; Xu et al., 2018; Yin et al., 2017; Zhang et al., 2018), the LOD of CsPb_0.95_Ni_0.05_Br_3_ NCs-based fluorescence sensor was much lower, probably contributing to the partial substitution and the uniform particle size (room-temperature synthesis method). Furthermore, when it came to the comparison with our previous study, although D_2_O-assisted FTIR spectroscopy provided an accurate and precise means of moisture analysis, it was easy to operate and is suitable for a variety of edible oils, but expensive FTIR equipment and certain sample pretreatment were required (Ye et al., 2023). The CsPb_0.95_Ni_0.05_Br_3_ NCs-based fluorescence sensor presented the properties of high sensitivity, fast response, and low-cost operation, which was particularly suitable for rapid field detection, but may be interfered with by other polar substances and required specific fluorescence detection equipment. In this context, the choice of method should be based on the detection needs, cost budget and operating conditions consideration.

### 3.3 Response mechanism of trace water in edible oils to CsPb_0.95_Ni_0.05_Br_3_ NCs

**Fig. 5:**
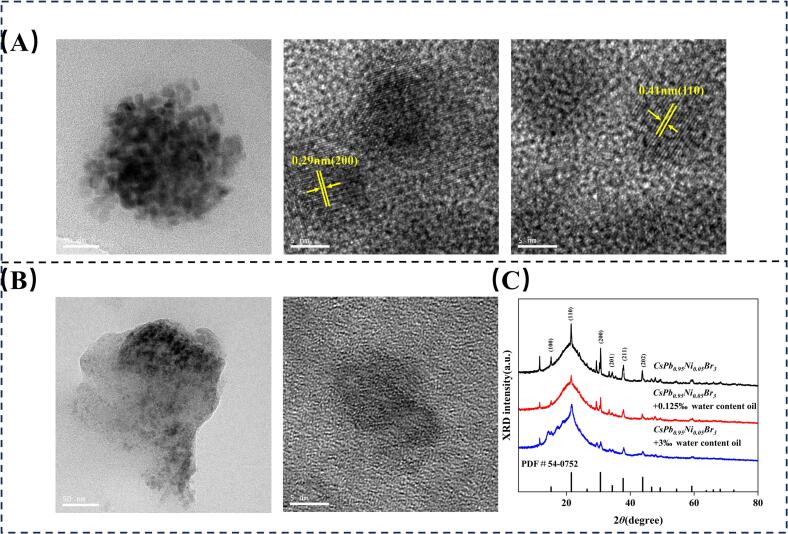
TEM images of CsPb_0.95_Ni_0.05_Br_3_ NCs after reaction with 0.125 ‰ WC (A) and 3 ‰ WC (B). XRD profiles (C) of CsPb_0.95_Ni_0.05_Br_3_ NCs after reacting with 0.125 ‰ WC and 3 ‰ WC.

To investigate the response mechanism of trace water in edible oils to CsPb_0.95_Ni_0.05_Br_3_ NCs, the microstructure and crystal structure of as-reacted CsPb_0.95_Ni_0.05_Br_3_ NCs exposed to low-WC and high-WC oils were analyzed by TEM and XRD. In contrast, *Soybean* oils with WC of 0.125 ‰ and 3 ‰ were selected as low-WC oil and high-WC samples, respectively. As shown in Fig. 5A, the particle size of CsPb_0.95_Ni_0.05_Br_3_ NCs reacted with low-WC oils was decreased and the lattice fringes were 0.29 nm and 0.41 nm, indicating that CsPb_0.95_Ni_0.05_Br_3_ NCs were partially dissolved and destroyed, compared with original CsPb_0.95_Ni_0.05_Br_3_ NCs (Fig. 1F). In terms of CsPb_0.95_Ni_0.05_Br_3_ NCs reacted with high-WC oils, the particle size further decreased and lattice fringes disappeared, demonstrating the amorphous structure and severe dissolution of CsPb_0.95_Ni_0.05_Br_3_ NCs (Fig. 5B). XRD characterizations were conducted to investigate the crystal structure of CsPb_0.95_Ni_0.05_Br_3_ NCs further. Fig. 5C performed that diffraction peaks (15.19°, 21.55° and 30.64°) of CsPb_0.95_Ni_0.05_Br_3_ NCs remained unchanged, while specific peak intensities decreased with the increase of WC, indicating that the crystal structure of CsPb_0.95_Ni_0.05_Br_3_ NCs was gradually destroyed by high-polar water (Acharya et al., 2024; Liu et al., 2021; Wang et al., 2022) In summary, the presence of trace water in edible oils can lead to the structure destruction and dissolution of CsPb_0.95_Ni_0.05_Br_3_ NCs, which in turn formed lattice defects and then decreased the fluorescence quantum efficiency, resulting in fluorescence quenching.

### 3.4 Practical applications to real edible oils

The response performances of the CsPb_0.95_Ni_0.05_Br_3_ NCs-based method to WC in various edible oils were investigated to verify the practical feasibility. Commonly, it was well known that primary fatty acids of edible oils were stearic acid, oleic acid, linoleic acid, and linolenic acid. According to the alterations of fatty acid compositions, monounsaturated fatty acid oils (*Camellia* oil, *Olive* oil) and polyunsaturated fatty acid oils (*Soybean* oil, *Flaxseed* oil) were selected. In particular, practical edible oil samples were obtained by spiking trace water. Overall, the recovery rate ranged from 96.3 % to 130 %, with an average RSD of 2.06 % (Table 1). For low-WC edible oils, RSD was relatively high in the range of 0.9 % to 7.1 %, and the recovery rate was relatively high in the range of 126 % to 130 %. It was believed that over-high recovery rates and high errors were attributed to the low WC in edible oils and low-abundant oil concomitants (such as polyphenols, VE, sterols). In terms of high-WC edible oils, RSD range was 0.1 %–2.0 %, and the recovery rates were 96.33 %–100.67 %, indicating an excellent detection performance. When it came to various edible oils, average RSD of two polyunsaturated fatty acid oils (*Soybean* oil 1.5 % and *Flaxseed* oil 2.03 %) was lower than that of monounsaturated fatty acid oils (*Camellia* oil 2.7 % and *Olive* oil 3.06 %), probably attributing to the standard curve established by polyunsaturated fatty acid oil (*Soybean* oil).

**Table 1:** Practical performance of CsPb_0.95_Ni_0.05_Br_3_ NCs-based method to various spiked edible oils.

Samples	Marking detection	CsPb_0.95_Ni_0.05_Br_3_ NCs-based fluorescence sensor			
	WC (‰)	WC (‰)	RSD (%)	Average RSD (%)	Recovery (%)
*Soybean* oil 1	0.50	0.63 ± 0.01	0.9	1.5	126
*Soybean* oil 2	2.00	2.02 ± 0.1	2.6		101
*Soybean* oil 3	3.00	3.02 ± 0.05	1.0		100.67
*Flaxseed* oil 1	0.50	0.64 ± 0.06	2.1	2.03	128
*Flaxseed* oil 2	2.00	1.96 ± 0.09	3.0		98
*Flaxseed* oil 3	3.00	2.89 ± 0.07	2.0		96.33
*Camellia* oil 1	0.50	0.63 ± 0.05	3.3	2.67	126
*Camellia* oil 2	2.00	1.94 ± 0.06	3.5		97
*Camellia* oil 3	3.00	2.95 ± 0.02	1.2		98.33
*Olive* oil 1	0.50	0.65 ± 0.04	7.1	3.06	130
*Olive* oil 2	2.00	2.17 ± 0.04	2		108.5
*Olive* oil 3	3.00	3.02 ± 0.05	0.1		100.67

**Fig. 6:**
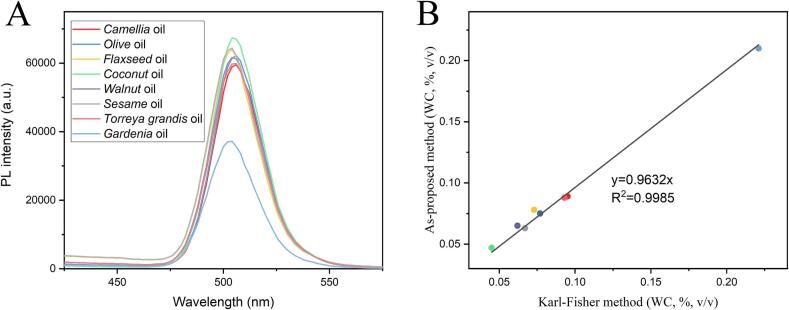
PL spectra of CsPb_0.95_Ni_0.05_Br_3_ NCs exposed to various commercial edible oils (A); the fitting curve of WC in real edible oils between the carl-fischer method and as-proposed CsPb_0.95_Ni_0.05_Br_3_ NCs-based method (B).

To evaluate the accuracy and practical feasibility of the as-proposed CsPb_0.95_Ni_0.05_Br_3_ NCs-based method further, 0.22 mL various commercial edible oils were mixed with 1 mL 1 mg/mL CsPb_0.95_Ni_0.05_Br_3_ NCs suspension. FL spectra were recorded after incubating at room temperature for 10 min, displayed in Fig. 6A. The result presented that peak positions remained unchanged within various edible oils while peak intensities varied significantly. According to the standard curve in Fig. 3B, WC in edible oils was calculated. With the comparison, Carl-Fischer titration method was used to evaluate WC in various commercial edible oils, as the actual value, shown in Table 2. The result demonstrated that WC in commercial edible oils was pretty low (about 0.7 ‰ (*v*/v)), meeting to the official range. Meanwhile, WC obtained from the as-proposed CsPb_0.95_Ni_0.05_Br_3_ NCs-based method was consistent with the Carl-Fischer titration method, with average relative errors of 5.18 %. Furthermore, a fitting curve of WC in various commercial edible oils between the Carl-Fischer method and as-proposed CsPb_0.95_Ni_0.05_Br_3_ NCs-based method was presented in Fig. 6B with an R^2^ of 0.9985, demonstrating superior sensing performance in practical edible oils. Thus, it was concluded that the as-proposed CsPb_0.95_Ni_0.05_Br_3_ NCs-based method was feasible and accurate for evaluating the WC in real edible oils.

**Table 2:** Practical performance of CsPb_0.95_Ni_0.05_Br_3_ NCs-based method to various commercial edible oils.

Oil types	Carl-Fischer method	As-proposed method	
	WC (‰, v/v)	WC (‰, v/v)	Relative error (%)
*Camellia* oil	0.95 ± 0.11	0.89 ± 0.17	6.32
*Olive* oil	0.77 ± 0.09	0.75 ± 0.18	2.60
*Flaxseed* oil	0.73 ± 0.10	0.78 ± 0.20	6.85
*Coconut* oil	0.45 ± 0.08	0.47 ± 0.19	4.44
*Walnut* oil	0.62 ± 0.06	0.65 ± 0.22	4.84
*Sesame* oil	0.67 ± 0.09	0.63 ± 0.19	6.00
*Torreya grandis* oil	0.93 ± 0.12	0.88 ± 0.23	5.38
*Gardenia* oil	2.21 ± 0.15	2.10 ± 0.20	5.00
*Average*			5.18

## 4 Conclusions

In this study, the FL intensity of CsPbBr_3_ NC was increased by Ni^2+^ substituting to Pb^2+^, and a sensitive and reliable fluorescence sensor was established. The result found that CsPb_0.95_Ni_0.05_Br_3_ NCs possessed considerable fluorescence properties and homogeneous crystalline structure. A negative leaner standard curve (R^2^ = 0.9969) was established between the FL intensity of CsPb_0.95_Ni_0.05_Br_3_ NCs and WC in edible oils with a LOD of 0.042 ‰. It was believed that trace water induced crystal structure decomposition by reducing the surface ligand concentration, partially replacing the surface ligand or completely destroying the surface ligand, thus inducing particle aggregation and lattice fringe disappearance. Furthermore, the CsPb_0.95_Ni_0.05_Br_3_ NCs-based fluorescence sensor can be used to evaluate WC of actual edible oils with a good linear relationship and satisfactory recovery rates. It had certain enlightenment significance for the rapid detection of edible oil quality, and can provide reference for developing more novel and effective detection methods in the future.

## CRediT authorship contribution statement

**Qin Ye:** Writing – original draft, Visualization, Investigation. **Penghao Zhu:** Writing – original draft, Investigation, Formal analysis. **Xianghe Meng:** Software, Resources, Methodology. **Jie Li:** Supervision, Methodology, Data curation. **Yuanchao Lu:** Writing – review & editing, Supervision, Methodology, Funding acquisition, Formal analysis.

## Declaration of competing interest

The authors declare that they have no known competing financial interests or personal relationships that could have appeared to influence the work reported in this paper.

## Data Availability

Data will be made available on request.
